# New avenues for phase matching in nonlinear hyperbolic metamaterials

**DOI:** 10.1038/srep08983

**Published:** 2015-03-11

**Authors:** C. Duncan, L. Perret, S. Palomba, M. Lapine, B. T. Kuhlmey, C. Martijn de Sterke

**Affiliations:** 1 Centre for Ultrahigh bandwidth Devices for Optical Systems (CUDOS), School of Physics, University of Sydney, NSW 2006, Australia; 2 Institute of Photonics and Optical Science (IPOS), University of Sydney, NSW 2006, Australia; 3 School of Mathematical Sciences, University of Technology Sydney, NSW 2007, Australia; 4 ITMO University, Saint Petersburg 197101, Russia

## Abstract

Nonlinear optical processes, which are of paramount importance in science and technology, involve the generation of new frequencies. This requires phase matching to avoid that light generated at different positions interferes destructively. Of the two original approaches to achieve this, one relies on birefringence in optical crystals, and is therefore limited by the dispersion of naturally occurring materials, whereas the other, quasi-phase-matching, requires direct modulation of material properties, which is not universally possible. To overcome these limitations, we propose to exploit the unique dispersion afforded by hyperbolic metamaterials, where the refractive index can be arbitrarily large. We systematically analyse the ensuing opportunities and demonstrate that hyperbolic phase matching can be achieved with a wide range of material parameters, offering access to the use of nonlinear media for which phase matching cannot be achieved by other means. With the rapid development in the fabrication of hyperbolic metamaterials, our approach is destined to bring significant advantages over conventional techniques for the phase matching of a variety of nonlinear processes.

Nonlinear frequency conversion is a technique to generate electromagnetic radiation at frequencies not present in the incident field^1^,^2^. An example is *second harmonic generation* (SHG) whereby a fundamental frequency (FF) *ω* is doubled via the interaction with a nonlinear medium^2^. Since the phase of the SH wave, generally, does not have the same position dependence as that of the FF wave, SH light generated at different positions may interfere destructively. Therefore, a phase matching condition must be satisfied so the SH fields add in phase. For SHG, it requires that *k*_2*ω*_ − 2*k_ω_* = 0, where *k_ω_*_,2*ω*_ are the wavenumbers of the FF and SH. Since *k* = *nω*/*c*, with *n* the refractive index and *c* the speed of light in vacuum, this requirement is equivalent to *n*(2*ω*) = *n*(*ω*).

Generally, phase matching requirements are not satisfied; for normal dispersion, for instance, *n*(2*ω*) > *n*(*ω*). In *quasi-phase matching* (QPM), the nonlinear properties are made to vary periodically, typically by reversing the sign of the nonlinear coefficient, so that efficient frequency conversion can be achieved^3^. However, the periodic modulation of crystal properties required for QPM, usually achieved by *poling*, is often challenging and can be used only for a limited number of materials^3^. Alternatively, in *birefringent phase-matching*, the refractive index differences due to dispersion is balanced by that between the ordinary and extraordinary wave in a birefringent medium, typically by appropriately choosing the propagation direction in the crystal. The birefringence can either be natural or can be due to form birefringence^4^,^5^. However, the birefringent phase matching requires that the difference between the extraordinary and ordinary refractive indices for the FF and SH must be larger than that due to dispersion. Since the classical birefringence is typically small, this method cannot be exploited in arbitrary nonlinear media.

The advent of metamaterials — artificially engineered materials with exotic properties — has opened wide opportunities for nonlinear optics^6^, offering novel approaches for phase matching^7^. These include the use of metamaterials with dual resonances, matched for SHG^8^,^9^; generation in reflection, exploiting negative refractive indices^10^,^11^,^12^,^13^,^14^; dispersion engineering in arrays and transmission lines^15^,^16^,^17^; as well as boosting conventional QPM techniques^18^,^19^.

Here, we systematically explore the novel opportunities towards a birefringent-like phase matching in *hyperbolic metamaterials*, or indefinite media, materials which behave like a metal in one direction but like a dielectric in another^20^,^21^,^22^,^23^. Implementations of hyperbolic metamaterials are available as alternating layers of metal and dielectric^24^, which behaves like a metal in the direction parallel to the layers and as a dielectric orthogonal to it, or as wire media^25^ or plasma wires^26^ in a dielectric background, which behave like a metal in the direction of the wires and like a dielectric orthogonal to them. For these composites to mimic uniform media, the transverse dimensions of the structural elements need to be much smaller than the wavelength of the radiation.

Certain nonlinear effects in hyperbolic metamaterials have been addressed already^27^,^28^,^29^,^30^,^31^, with emphasis on non-local enhancement^27^, power-dependent transmission^28^, polarisation switching^29^ and all-optical modulation^30^. In particular, SH processes were theoretically and numerically analysed^31^ for layered hyperbolic metamaterials implemented with silver layers and metal oxides; assuming a point-dipole excitation near the surface of such material, the authors predicted a formation of double-resonance cones and discussed their implications on SH imaging^31^. Nonetheless, a systematic investigation of the various phase-matching opportunities in hyperbolic metamaterials was so far not undertaken.

Here we argue that unusual dispersion in layered hyperbolic metamaterials, see Fig. 1(a), provides a number of promising means to realise phase matching. It is true that in the typical implementations of layered hyperbolic media the dissipation is relatively high due to the presence of the metal. Nonetheless, our aim here is to investigate whether or not it is worth considering hyperbolic media for purposes of phase matching. In most of this paper we neglect losses so as to be able to concentrate on the phase matching aspects. However, we give an example showing that the dissipation length can be larger than the beat length of the FF and SH, thus demonstrating that the novel dispersion of layered metamaterials may be exploited for phase matching of SHG.

In hyperbolic media, the isofrequency surface, which maps out all allowed **k**-vectors for a given frequency, is hyperbolic, see Fig. 1(b), whereas in naturally occurring birefringent media it is elliptical. This allows, in principle, for propagating waves with arbitrarily large wavenumbers. If a material is hyperbolic at the fundamental frequency, the large wavenumbers accessible at that frequency should guarantee the possibility of compensating for any variation in refractive index due to dispersion at the second harmonic frequency. However, hyperbolic materials are highly dispersive, in particular at optical frequencies, so their practical implementation requires a more detailed and careful analysis.

This paper is organised as follows. First, we carry out a systematic analysis of the frequency-dependent shape of the normal surfaces of layered hyperbolic media using homogenisation, which ignores the spatial dispersion in these media. We then analyse all possible combinations of polarisation for elliptic and hyperbolic regimes that can occur at the FF and SH. From this we identify a small subset of configurations for which phase matching is achievable. Next, we use a rigorous transfer matrix method to confirm that phase matching is achieved beyond the effective medium approximation. Finally, we provide a realistic example with practically available materials, and show that phase matching is feasible in spite of a noticeable dissipation, and even when the dispersion would not be sufficient for a classical birefringent scheme.

## Results

### Normal surfaces of layered media

**Figure 1:**
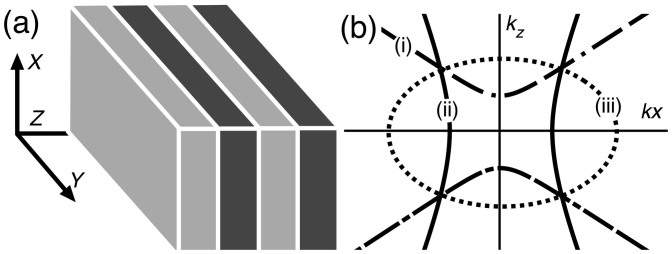
(a) Schematic of a layered hyperbolic medium with coordinate axes; (b) isofrequency surfaces showing: (i) north-south (NS) and (ii) east-west (EW) hyperbolic; (iii) elliptical.

For the layered geometry shown in Fig. 1(a), and defining the z-axis of our cartesian coordinate system to be perpendicular to the layers, the permittivity tensor 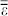 is diagonal with the components
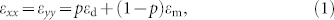

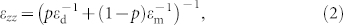
provided 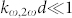, with *d* the period. Here *ε*_m,*d*_ are the permittivities of the metal and dielectric respectively, and *p* is the dielectric volume fraction. According to the standard theory for plane waves in uniaxial media, for *ordinary* (TE) waves, 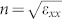 and for *extraordinary* (TM) waves,
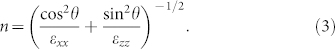
where cos *θ* = *k_z_*/||**k**||. This equation may be cast in the form
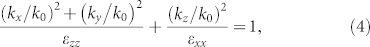
where *k*_0_ = *ω*/*c*. The solution set in **k**/*k*_0_ of Eq. (4) represents the surface formed by revolving a conic section about the *z*-axis, which suggests the threefold typology illustrated in Fig. 1(b): borrowing the geometers' nomenclature, a normal surface is (i) *north-south* hyperbolic (NS) if *ε_xx_* > 0 > *ε_zz_*; (ii) *east-west* hyperbolic (EW) if *ε_zz_* > 0 > *ε_xx_*; and (iii) elliptical if all diagonal components are positive. The medium is metallic if all diagonal elements are negative. The metamaterial literature also applies the term *cut*-*off* to elliptical media and *anti*-*cutoff* to EW. For our purposes, it is necessary to introduce the third category of NS: to give one example of an important difference between NS and EW media, ordinary waves propagate in the latter but not the former. It is also desirable to use nomenclature that draws attention to the shape of the medium's normal surfaces, since our problem ultimately reduces to that of finding intersections of these surfaces. Matching extraordinary FF with extraordinary SH is possible because normal surfaces of different types do intersect, which we demonstrate presently.

**Figure 2:**
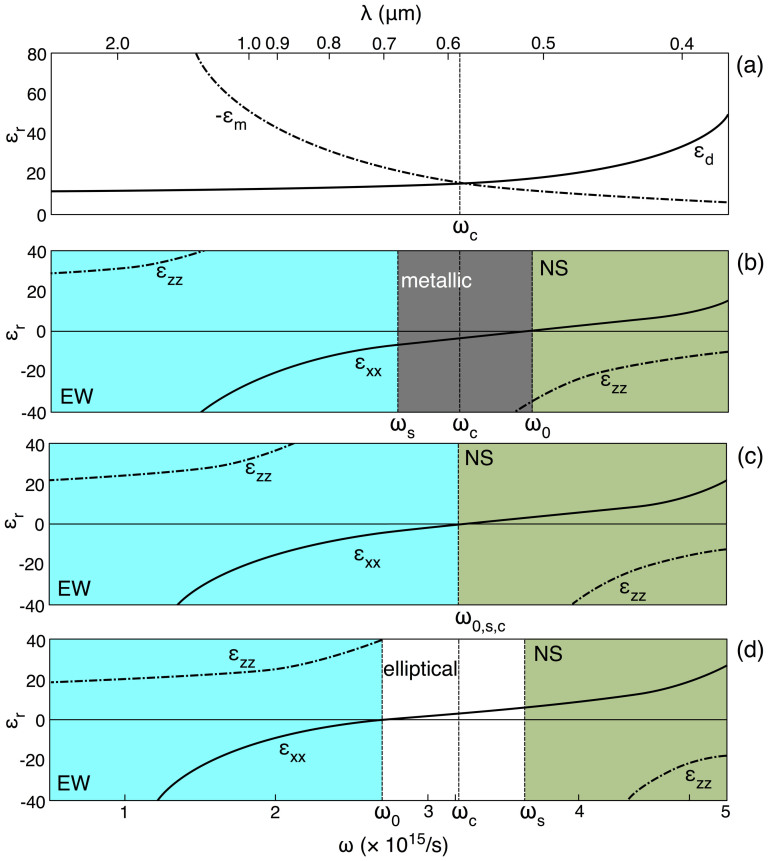
Dispersion of the permittivities of (a) GaAs (*ε*_d_; Ref. 34) and Au (*ε*_m_; Drude model with plasma frequency 2.1 × 10^15^ Hz), compared with *ε_xx_* and *ε_zz_* of a layered medium containing (b) 40%, (c) 50% and (d) 60% dielectric.

The same combination of dielectric and metal may exhibit normal surfaces of all three types, depending on frequency, as Figs. 2(b)–(d) illustrate. The figures show how the dispersion relations of homogenized layered media with different fill fractions vary with frequency. The dispersion of the constituents, taken here to be GaAs and gold, is shown in Fig. 2(a). Anomalous dispersion is ignored in calculating *ε*_d,m_ in order to make Fig. 2 represent clearly the qualitative features that are common to all normally dispersive materials. We thus assume, for simplicity, that (i) *ε*_m_ and *ε*_d_ are normally dispersive; and, in addition, that (ii) *ε*_d_ > 0 > *ε*_m_ at all frequencies. It follows from Eqs. (1) and (2) that in general a layered medium is EW at low frequencies, NS at high frequencies and elliptical or metallic in the intermediate range between these two. To formalise the meaning of “low”, “intermediate” and “high” in this context, the figure marks three defining frequencies: the *critical frequency* *ω*_c_ where *ε*_d_ = −*ε*_m_, the *singular frequency* *ω*_s_ at which *ε_zz_* diverges, and *ω*_0_, where *ε_xx_* = 0. The medium is NS when *ω* > *ω*_s_, *ω*_0_, EW when *ω* < *ω*_s_, *ω*_0_, and elliptical when *ω*_0_ < *ω* < *ω*_s_. Whether the medium behaves elliptically or like a metal in the intermediate regime depends on the fill fraction of dielectric (*p*), a dependence shown in the progression of Fig. 2, (b)–(d). If *p* < 50% then *ω*_s_ < *ω*_c_ < *ω*_0_ and thus the intermediate regime is metallic, while *p* = 50% implies *ω*_c_ = *ω*_s_ = *ω*_0_ and hence that there is no intermediate regime, and *p* > 50% implies that *ω*_0_ < *ω*_c_ < *ω*_s_ and the intermediate regime is elliptical.

### Phase matching in layered media

#### Homogenization regime

**Figure 3:**
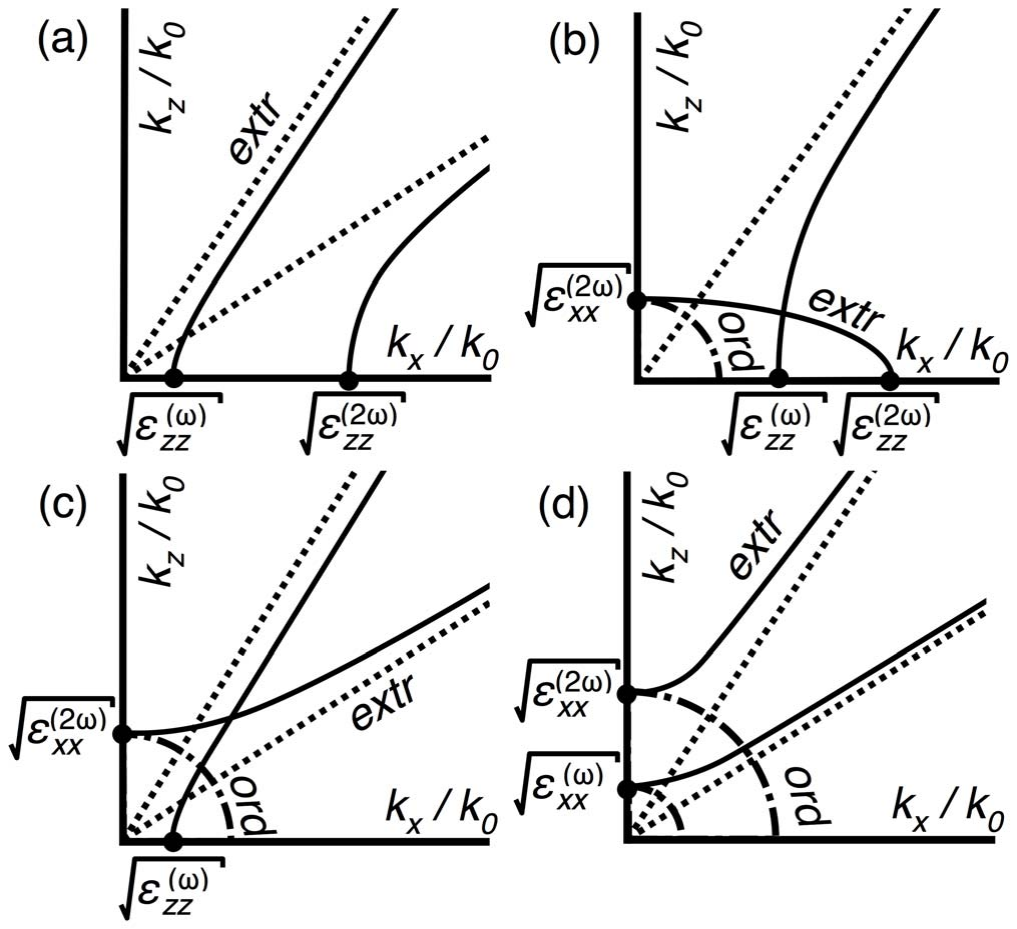
Isofrequency plots for the four cases (a)–(d) as summarised in Table 1; (a) solid lines show the limbs of FF and SH EW hyperbolae and dotted lines their linear asymptotes; (b) solid lines show hyperbolic FF and elliptical SH, and the dash dotted line the circular SH ordinary mode; (c) same as (a), but with SH hyperbola NS, and ordinary mode shown dash dotted; (d) same as (a) but with both hyperbolae NS, and ordinary modes shown dash dotted.

The results from the above section restrict the possibilities of hyperbolic phase matching to four cases, schematised in Table 1 and illustrated in Fig. 3. A fifth case (elliptical FF with NS SH) can be eliminated because the minimum phase index at the SH exceeds the maximum at the FF. We show below that solutions to the phase matching condition must exist for some *θ* in either (a) or (c), as well as in cases (b) and (d) for any combination of normally dispersive dielectric and metal.

**Cases (a) and (c):** In these cases we seek general conditions under which two hyperbolic normal surfaces intersect. Noting that a hyperbola is asymptotically a straight line passing through the origin, the hyperbolae intersect if and only if the limb that approaches its asymptote from below has the steeper gradient. It follows from Eq. (4) that the gradients of the asymptotes are given by 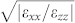 and so the necessary and sufficient condition for the existence of an extraordinary-extraordinary matching angle is
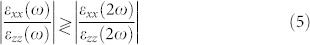
with “<” for (a), and “>” for (c). The right-hand side of Eq. (5) is guaranteed to vanish when 2*ω* → *ω*_0_, with the left hand side remaining positive, and it is possible to make this approach from above in the NS SH regime. In contrast, it is not possible to make the left hand side vanish in a similar way and remain in the EW SH regime. A second solution in case (c) involves the ordinary SH mode, which is not possible in case (a) because ordinary modes do not propagate in EW media.

**Case (b):** We now seek general conditions for the intersection between hyperbolic FF and elliptical SH normal surfaces. We make use of three facts: that the difference between the FF and SH phase indices varies continuously with propagation direction; that the phase index of the FF extraordinary mode is bounded from below but unbounded from above; and that the phase index of the SH extraordinary mode is bounded from above. We write 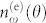 and 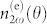 for the extraordinary phase indices of the FF and SH respectively at angle *θ* from the *z*-axis, and let *θ_a_* be the angle between the EW asymptote and the *z*-axis (*i.e.*, the angle at which Eq. (2) is singular). The assumption that the constituents are normally dispersive implies, on differentiating Eq. (2) with respect to *ω*, that
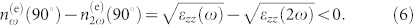
Further, from Eq. (3), as *θ* → *θ_a_*,
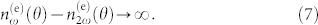
The intermediate value theorem implies that for some *θ*_0_, *n_ω_*(*θ*_0_) = *n*_2*ω*_(*θ*_0_). Examining Fig. 3(b), the existence of a solution is intuitive on noting the connection between the *k_x_* intercepts and the dispersion of the medium.

**Case (d):** The final case we consider involves matching an NS FF with an NS SH and differs from case (a) in that advantage may be taken of propagating ordinary modes. The condition on the existence of a phase matching angle is that the minimum phase index of the FF extraordinary mode is less than the uniform phase index of the ordinary SH. Eq. (4) shows that for NS (*ε_xx_* > 0) the lower bound on *n* is 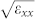. For the SH ordinary mode, 
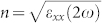
. The function *ε_xx_*, being the arithmetic mean of two monotonic increasing functions, is also monotonic increasing, that is, 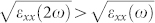, and so a matching angle is guaranteed to exist.

#### Beyond the effective medium approximation

The homogenisation in Eqs. (1)–(2) assumes that the effective 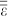 is independent of **k**, and we now present a Kronig-Penney model that relaxes this assumption, in order to confirm the results obtained from homogenization and to perform numerical calculations. This approach also allows us to include the effects of dissipation. Working in the same coordinate system as in Fig. 1, we treat the structure as infinitely periodic, which implies that the Bloch condition holds, **E**(*z* + *d*) = exp(*ik_z_d*)**E**(*z*) with *k_z_* the Bloch vector and *d* the spatial period. In this section we concentrate on the most interesting case where both the FF and SH are extraordinary waves. Knowing the boundary conditions imposed by Maxwell's equations at the interface between metal and dielectric gives us, through the matrix transfer method, a second relation between **E**(*z* + *d*) and **E**(*z*), which together with the Bloch condition, allows *k_z_* to be found in terms of *k_x_* and *ω* according to the equation^32^:
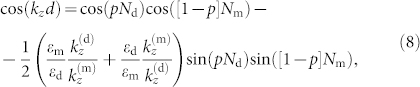
with 
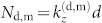
 and 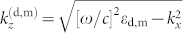. As 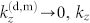 approaches the value predicted by homogenisation. Numerical comparison showed the discrepancy to be less than 2% when 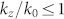.

**Figure 4:**
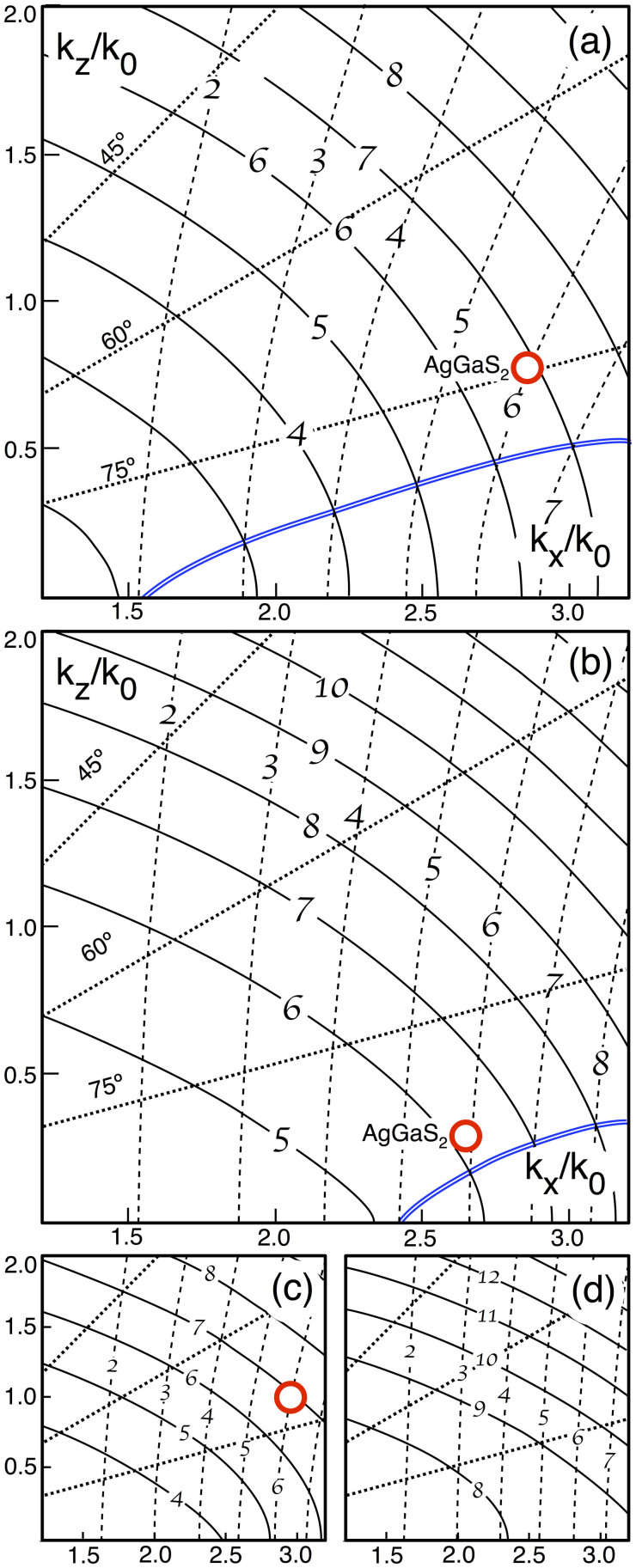
(a) FF (1064 nm) and SH normal surfaces in *k*-space for a medium with *d* = 100 nm, *p* = 0.85 and *ε*_m_ taken from tabulated values for silver^35^. Each dashed curve corresponds to a value of the variable *ε*_d_(*ω*) and likewise solid curves *ε*_d_(2*ω*), as indicated by the calligraphic numerals. Lines of constant angle to the normal are shown dotted. The matching angle solution for AgGaS_2_ is circled^33^. The double solid blue line shows the curve *ε_d_*(*ω*) = *ε_d_*(2*ω*). (b) the same for FF at 1550 nm; (c) same as (a) with the fill fraction *p* = 0.7; (d) same as (b) with *p* = 0.75.

To plot the dependence of solutions to Eq. (8) on the material parameters *ε*_d,m_, we fix *ε*_m_ and the geometrical parameters *d* and *p*, and let *ε*_d_ be a free variable. For our purposes, we require *ε*_d_ at the FF and SH. The *k* space is also effectively two dimensional, since the solutions are axially symmetric about *k_z_*. Hence, Eq. (8) maps from coordinates in one plane, the space of values of *ε*_d_, to another, *k* space, a mapping which we can visualise by plotting *ε*_d_ coordinate curves in cartesian *k* space, as in Figs. 4(a)–(d). In these figures, the dashed curves labelled with calligraphic numerals indicate constant FF *ε*_d_ and the solid curves constant SH *ε*_d_. Each curve plotted represents a unit increment. The rectilinear axes show the *k* solution corresponding to a given permittivity coordinate. Dashed diagonal lines indicate the matching angle with respect to the *z*-axis. The metal constituent is silver, with permittivities taken from tabulated values. Panels (a) and (c) take the FF to be 1064 nm, while (b) and (d) likewise 1550 nm. Panels (a) and (b) set the fill fraction *p* = 0.85, whereas in panels (c) and (d), *p* = 0.75. One may read off solutions for any choice of dielectric; the figures represents Eq. (8) in complete generality in this respect.

As an illustrative example known in the literature^33^, we select AgGaS_2_ which has 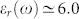 for FF 1064 nm, and 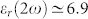 at the SH. By locating the coordinate (6.0, 6.9) in *ε* space, Fig. 4(a) shows the matching solution to be 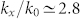, 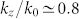 (circled), with matching angle 75°. The double solid curve shown in Fig. 4(a) marks all points where the FF and SH permitivitties are equal, providing an upper bound on matching angles. In contrast to elliptical media, the more dispersive the dielectric, the closer the matching angle is to the normal. Again, the phase matching obtained here is between extraordinary waves for both, FF and SH, and is thus not birefringent phase matching.

Fig. 4(b) is similar to Fig. 4(a) but is for a FF of 1550 nm, leaving all other parameters to be the same. This changes the metal permittivities from *ε*_m_(*ω*) = −58, *ε*_m_(2*ω*) = −12 at 1064 nm, to *ε*_m_(*ω*) = −129, *ε*_m_(2*ω*) = −29 at 1550 nm. Comparison with Fig. 4(a) reveals two significant effects of spatial dispersion. First, below a certain value of the dielectric permittivity, the SH modes become evanescent in the *z* direction, that is, *k_z_* becomes purely imaginary. This effect must be due to spatial dispersion because simple homogenisation predicts evanescent waves only when *p* < 0.5 and when the magnitudes of *ε*_d,m_ are of the same order (see Fig. 2(b)), neither of which holds here. The occurrence of this effect depends on the metal permittivity: while in Fig. 4(a) modes propagate at dielectric permittivities as low as *ε*(2*ω*) ≈ 2, in Fig. 4(b) modes are already evanescent when *ε*(2*ω*) ≈ 4.5. Moreover, the *k_z_* of all contours is lower in (b) compared to (a), and the contours are more densely spaced.

The second effect of spatial dispersion is that the FF normal surfaces only weakly depend on *k_x_* as the FF dielectric permittivity decreases, an effect which is particular striking in Figs 4(c) and (d). This effect too becomes more pronounced the more negative the metal permittivity is, born out by comparing the slopes of the dashed FF curves in Figs. 4(a) and (b).

Figures 4(c) and (d) are similar to 4(a) and (b), but with a reduced dielectric fill fraction. Comparing Figs. 4(a) and (c), decreasing the fill fraction transforms higher permittivity SH curves from ellipses into NS hyperbolae, as the simple homogenisation model predicts. The evanescence threshold observed in Figures 4(b) is increased in Fig. 4(d) from between 4.5 and 5 to between 7.5 and 8.

Though a systematic treatment of dissipation is not performed here, we have calculated the propagation length, the length over which the field amplitude decays to 1/*e* of its initial value, in the particular case of a layered medium composed of 15% Ag and 85% AgGaS_2_ with a spatial period of *d* = 100 nm and a fundamental wavelength *λ*_FF_ = 1.55 *μ*m. AgGaS_2_ is negatively birefringent at these wavelengths but insufficiently so for conventional phase matching, and in performing our calculations we make the approximation that 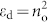, where *n*_o_ is the ordinary refractive index. The propagation length of the FF mode is 17.1 *μ*m and of the SH, 14.4 *μ*m. It is instructive to compare these numbers with the coherence length *L* in AgGaS_2_ at 1.55 *μ*m, computed according to the formula^3^
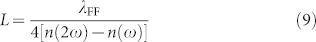
With the reported data^33^, for ordinary FF and extraordinary SH, *n*_e_(2*ω*) − *n*_o_(*ω*) = 0.02 (subscripts e and o denote the extraordinary and ordinary index), so that *L*_eo_ = 19.4 *μ*m. Taking ordinary FF and ordinary SH, *L*_oo_ = 5.4 *μ*m. The propagation length of the layered structure improves on *L*_oo_ and is comparable to *L*_eo_. This example shows that frequency conversion can be achieved prior to the decay of the signals due to dissipation.

## Discussion

In conclusion, we propose and systematically investigate the use of hyperbolic dispersion for phase matching nonlinear frequency generation. Our approach provides an alternative for efficient phase matching, overcoming certain limitations of the two classical techniques. A particular benefit of the new method is that the dispersion can be designed independently of nonlinear properties, therefore expanding phase matching opportunities towards, in principle, arbitrary nonlinear materials.

Remarkably, amongst the many different combinations of normal surfaces at the FF and SH frequencies, we conclude that only four permit phase matching for SHG. Appropriate structures can have low metal volume fractions, thus promising modest absorption. Provided that these absorptive losses can be limited to acceptable levels, layered metamaterials thus provide a route to engineering materials for phase matching of materials without intrinsic birefringence. Figs. 4 are a convenient way to represent the phase matching conditions. Using this figure, we find that a key difference with conventional (elliptical) media is that hyperbolic normal surfaces enable phase matching when all waves are extraordinary. This allows for the use of the diagonal elements of the *χ*^(2)^ tensor, which tend to be larger than the off-diagonal elements^2^.

While a systematic investigation of the dissipation is beyond the scope of this paper, we presented a realistic example in which the decay length of the fields exceeds the beat length of the FF and the SH. Future work would need to consider the details of the SHG process, *i.e.*, the particular element of the *χ*^(2)^ tensor which is being exploited, the strength of the interaction, the coupling geometries for the two frequencies, as well as other nonlinear processes which require phase matching.

We note waveguides made with hyperbolic materials can carry positive and negative group velocity modes depending on frequency^36^,^37^,^38^,^39^,^40^. This may lend itself for backward propagating SHG, which can be highly efficient^41^. The detailed study of SHG possibilities in such configuration however is beyond the scope of this paper.

Though we considered phase matching for second-harmonic generation, other frequency conversion processes, all of which have phase matching conditions which take the form of a relation between the refractive indices at the different frequency involved in the process, can similarly benefit from the dispersion afforded by hyperbolic metamaterials. The rapid progress in the design and fabrication of hyperbolic media provides the confidence that hyperbolic phase matching will be able to be applied to a range of nonlinear materials and materials where conventional method cannot be used, enabling further progress in nonlinear optics.

## Author Contributions

Theoretical analysis was performed by C.D. with the help and advice from M.dS., B.K. and M.L. and numerical simulations were provided by L.P. and C.D. The results were discussed and the manuscript was prepared jointly by all the authors.

## Figures and Tables

**Table 1:** The four different possibilities which may lead to phase matching of SHG in layered metamaterials

Case	FF	SH
(a)	EW	EW
(b)	EW	elliptical
(c)	EW	NS
(d)	NS	NS
